# First record of an adult female ring-tailed lemur (*Lemur catta*) making a male-specific howl vocalization

**DOI:** 10.1007/s10329-026-01270-3

**Published:** 2026-07-14

**Authors:** Lisa M. Paciulli, J. L. Verdolin, Diego Garza Guajardo, Carmen M. Cromer

**Affiliations:** 1 Department of Biological Sciences, North Carolina State University, Raleigh, NC USA; 2 Research PackTrack Program, Department of Biological Sciences, North Carolina State University, Raleigh, NC USA; 3 Department of Natural Sciences, SUNY Empire State University, Saratoga Springs, NY USA; 4 MedCenter Air Hospitals and Health Care, Charlotte, NC USA; 5 Department of Biology, Duke University, Durham, NC USA

**Keywords:** Vocalization, Primate, Communication, Sex differences

## Abstract

Vocal communication is a critical aspect of social life in group living species. Ring-tailed lemurs (*Lemur catta*) are one of the most studied lemur species, yet the quantity of male specific vocalizations remains unclear. Howling has previously been described as a male only vocalization. While investigating Duke Lemur Center (DLC: Durham, N.C.) ring-tailed lemurs’ responses to novel objects when separated from their group for five minutes, video and audio opportunistically captured a female howling. We sought to determine whether these vocalizations were acoustically similar to male howling or represented a different vocalization. We focused on body posture and acoustic structure of the call by analyzing spectrograms of the female’s calls, and compared these features to male howls. Body posture comparison revealed similarities in the female’s position and orientation of the body to males when howling. From the spectrograms, we compared the fundamental, maximum, maximum harmonic, dominant, supradominant frequencies, interharmonic intervals, and number of harmonics to values extracted from the published literature. A linear discriminant function analysis (DFA) failed to distinguish the female’s calls from male howls. This is the first recording and analysis of female howling vocalizations. While male ring-tailed lemurs are known to howl to advertise their location, it’s possible that female lemurs may howl for different reasons. Females may howl during high stress events, and we suggest future research to understand the conditions that elicit howling vocalizations in females in the wild and in captivity.

**Supplementary Information:**

The online version contains supplementary material available at https://doi.org/10.1007/s10329-026-01270-3.

## Introduction

Vocal communication is a critical aspect of social life in group living species. Nonhuman primate vocal communication, vocal repertoire complexity, and context vary widely across species (Bouchet et al. 2013; Fichtel and Kappeler 2022; McComb and Semple 2005). In the Suborder Strepsirrhini, there is tremendous cross-taxa variation in vocal repertoire size, especially among the Malagasy lemurs (Zimmerman 2017). Within all basal primates, ring-tailed lemurs (*Lemur catta*) have one of the largest vocal repertoires, with 22 distinct vocalizations having been identified (Macedonia 1993). Despite the recognition of a *female-dominated social structure* in ring-tailed lemurs (Lewis 2019), the majority of research examining vocal behavior has focused on males and juveniles (Bolt 2013a,b; Bolt 2014, 2020, 2021; Bolt et al. 2015; Bolt and Tennenhouse 2017; but see Petter and Charles-Dominique 1979; Macedonia 1986, 1990a, b, 1993; Oda 1996). The emphasis on male vocalizations has led to a critical gap in our understanding of the importance and context of female vocal behavior in ring-tailed lemur society.

Ring-tailed lemurs are arguably one of the most studied lemur species with the first studies occurring in the early 1900s (Pocock 1918) and becoming more frequent in the 1950s and 60s (e.g., Jolly 1966). Moreover, there are several long-term research sites such as the Beza Mahafaly Reserve, Madagascar at which ring-tailed lemurs have been studied for over 30 years (Gould 1996; Gould et al. 2003; Meredith 2018; Sauther 1989). Macedonia (1993), however, remains the most comprehensive compilation of ring-tailed lemur vocalizations in a semi-free ranging captive environment, including acoustic structure and context. Despite the importance of vocal communication in nonhuman primate social dynamics and evidence for sexual dimorphism in vocalizations across species (Aung et al. 2023), there is little information about male specific vocalizations (however, see male orangutan (*Pongo spp.*) long calls: Delgado 2007). In ring-tailed lemurs, however, howling has been identified as a male only vocalization used predominantly by adolescents and adult males, with females responding to howls with different vocalizations, such as wails and moans (Andrew 1963; Bolt 2013; Jolly 1966; Macedonia 1990a, b, 1993). Males howl at dusk as a chorus, they howl more during the breeding season, and/or when they are separated from the group (Bolt 2013; Jolly 1966; Macedonia 1990a, b, 1993; Petter and Charles-Dominique 1979).

The leading hypothesis is that male howling is used for mate defense in intergroup encounters (Bolt 2013). However, it has been noted that the purpose of male howling is independent of male rank and may function to communicate a group’s presence and location during such encounters (Bolt 2013; Jolly 1966; Macedonia 1993). Given the instability of male rank compared to female rank (Sauther et al. 1999) and the fact that howling may be involved in intergroup encounters, howling has previously been classified as a male-only vocalization. However, two previous anecdotal observations provide support for the idea that females are capable of howling.

Jolly (1966) documented the first possible howl of a female ring-tailed lemur at Beza Mahafaly Reserve. She heard a howl from up in a tree when no males were known to be in the direction of the call. However, Jolly (1966) cautiously noted that the howler was not in direct sight during the call, making the identity of the caller uncertain. Twenty-five years later, Sauther (1991) reported the second instance of a female howling, again at Beza Mahafaly. Sauther (1991) observed the alpha female of a troop of ring-tailed lemurs move 50 m away from her troop, climb a crook of a tree, and then howl twice before giving birth. Sauther (1991:470) remarked that a howl “has never been clearly observed in female *L. catta*” and that “No other female was observed to give this standard male *L. catta* vocalization.” Thus, the ambiguity of the sex on one occasion and the extreme circumstance of the other resulted in researchers concluding that these howling females were anomalies.

Here, we provide the first confirmed audio and video evidence of the ability of female ring-tailed lemurs to howl, and that the range of expression and usage of this vocalization is greater than previously documented. We compared the acoustic metrics of male howls reported in the literature to the recorded howl. We also analyzed the posture of the female during the call as well as the structure of the howl and compared acoustic measurements among different vocalizations.

## Methods

### Subject and opportunistic recordings

On April 6th, 2018, CMC opportunistically captured two howling vocalizations at the Duke Lemur Center (DLC) in Durham, North Carolina (NC) while conducting a study using responses to novel objects to assess ring-tailed lemur personality (Duke IACUC Protocol A218-16-10). During the study, a randomly selected ring-tailed lemur, a DLC-born 14 year old adult female named “Tellus,” was separated from her adult male companion, “Licinius” (Date of birth: 3/17/93). Tellus was born on March 18, 2004 into a social group of six ring-tailed lemurs living in a 14 acre natural habitat enclosure (NHE) with access to indoor and outdoor enclosures attached to a building. Until 2007, Tellus was in a group of ~ 10 individuals. Then, she was removed from this social group and paired with Licinius. For eight years, the two were housed inside with access to a small caged-outdoor enclosure, but were not free-ranging. In 2015, Tellus and Licinius once again were free-ranging in an NHE with 7.4 acres with a family of four sifaka (*Propithecus coquereli*).

During the novel object personality study, Tellus and Licinius were indoors where they normally had access to multiple indoor enclosures, each approximately 270 square feet providing 2,700 cubic feet per animal (DLC 2026). During the first trial, Tellus and Licinius were separated into adjoining visible enclosures for five minutes. While the two were separated, a group of red-ruffed lemurs (*Varecia rubra*) located approximately 15 m down the hallway made a series of pulsed squawks, a high-amplitude call indicating extreme arousal, including when detecting a predator (Pereira 1988). The red-ruffed lemurs pulse squawk 8 to 15 times a day (E. Ehmke pers. comm.), and the vocalization often triggers a series of vocalizations from other lemur groups. In this instance, the pulsed squawk was followed by *V. rubra*, *V. variegata*, and *L. catta* groups vocalizing, resulting in a cacophony of vocalizations (CMC pers. obs.). During the ensuing commotion, the female Tellus vocalized twice when she was alone in the enclosure next to Licinius. CMC video- and audio-recorded Tellus’s body position and vocalizations with an iPhone 7 Plus phone (Apple^®^ 2016). Tellus participated in the novel object personality study five times, and this was the only time that this type of behavior was observed. In the other trials, Tellus behaved as the other female lemurs did by keeping a distance from the novel object. Licinius also participated in the study five times, and he behaved as the other males did, scent marking the enclosure.

### Posture analysis

Because the female’s appearance and calls seemed similar to the body posture of howling male ring-tailed lemurs previously recorded at the DLC, we decided to compare the female’s posture during the howls to descriptions of males howling in the published literature (Andrew 1963) and to those of DLC Adult Male 1, “Fritz.” To make the comparison, we used screenshots from the videos of the female Tellus and Male 1 while they were vocalizing. We evaluated the position of the body (vertical / upright or horizontal), head (facing up or facing down), neck (contracted or stretched), chest (expanded or collapsed), and whether or not the lemur was sitting or standing and holding a substrate.

### Vocal analysis

To determine whether the female’s vocalizations were similar to male howls, we compared three sets of vocal characteristics. First, we uploaded the recordings of Tellus’s two calls into the audio program Audacity^®^ (Audacity 2019). We generated spectrograms in Audacity^®^ (2019) with an 8 kHz sampling rate, 50% window overlap with 8 Hz frequency resolution, and 250 ms temporal resolution. Then, we compared the

Fundamental, Maximum, Maximum Harmonic, Dominant, and Supradominant Frequencies (all kHz), as well as the Interharmonic Intervals (kHz) and Number of Harmonics of female Tellus’s two spectrograms to the same characteristics in the spectrograms of 43 howls made by four males. Thirty-two of the howls came from DLC Male 1 (Fritz) recorded by CMC, one howl came from DLC Adult Male 2 (Licinius) recorded by CMC, eight were from Male 273 (Bolt 2013), and two were from Macedonia’s (1993) Male 1 images h and i. Second, we compared the start, end, and peak frequencies, and duration of Tellus’s two calls to these features in the two DLC males’ howls that CMC recorded plus 110 howls made by nine DLC ring-tailed lemur males that Macedonia (1993 Table 1A) recorded.

Third, we compared the overall visual characteristics of the striations on the female

Tellus’s spectrograms to the visual characteristics of the spectrogram striations of DLC

Male 1 (Fritz) and Male 2 (Licinius) and Macedonia’s howl #1 and howl #2 (Macedonia 1993). The number, straightness, duration, location, and darkness (intensity) of the striations were compared.

### Statistical analysis

The acoustic frequencies (kHz), as well as the interharmonic intervals (kHz) and number of harmonics of female Tellus’s two spectrograms were compared to the spectrograms of 43 howls made by three males. We evaluated Spearman’s rank correlations between parameters to determine which parameters to retain and which to remove from further analyses. This allowed us to remove highly correlated parameters to reduce or eliminate multicollinearity among the variables used for classification.

Of the seven vocal parameters considered, three were significantly correlated (*r* > 0.9) with the others (Supradominant Frequency (kHz), Interharmonic Intervals (kHz), and Number of Harmonics) and therefore, were removed from further analysis. We used the four remaining variables: Fundamental, Maximum, Maximum Harmonic, and Dominant frequencies in a Linear Discriminant Function Analysis (DFA) to determine whether Tellus’ calls could be acoustically differentiated from male howls. DFA classifies unknown individuals and the probability of their classification into a certain group. The howls were classified based on orthogonal linear functions generated by the four variables (Brunner and Giannini 2011). We used values of Wilks’ Lambda to estimate how strongly the acoustic variables of the calls contributed to the discrimination of howls between the female and the males. All statistical analyses were carried out in JMP v17.0 (SAS, 1989–2023).

## Results

When comparing the female body position when she vocalized to a male ring-tailed lemur’s body position when howling, both the female and the male lemurs’ bodies were in a vertical/upright position, their heads and necks were stretched and raised upward, their chests were expanded and pushed out, they were sitting on their haunches, and they were gripping a substrate. (See Suppl Figs. 1 A and 1B.)

Table 1 shows the start, end, and peak frequencies and durations of the female ring-tailed lemur’s two vocalizations compared to two DLC ring-tailed lemur male howls that we recorded, plus 110 howls of nine DLC males from Table 1A of Macedonia (1993). The median (which is also the average because there are only two numbers) start frequency of Tellus’s vocalizations was 844.5 Hz, lower than the median start frequencies of all of the other males’ howls (two DLC males = 1,148.5 Hz, nine wild male ring-tailed lemurs (110 howls) = 1040 Hz (Macedonia 1993). The median (and average because there are only two numbers) end frequency of Tellus’s vocalizations was 950 Hz, in between the median end frequencies of two DLC males’ howls (1049 Hz), and the 110 howls made by the nine male ring-tailed lemurs (880 Hz, Macedonia 1993).

Peak frequencies varied from a low of 928 Hz (Tellus, howl #1) to a high of 1171

Hz (Licinius). The nine males’ howls’ median peak frequency was close to Licinius at 1160 Hz. The median (and average) duration of Tellus’s call was 1.151 s, and the median duration of the DLC males’ howls was 1.1815 s. The 110 howls had a shorter median duration of 0.762 s.

**Table 1:** The start, end, and peak frequencies and durations of the female ring-tailed lemur’s two vocalizations compared to two DLC ring-tailed lemur male howls that we recorded, plus 110 howls of nine males from Table 1A of Macedonia (1993)

Ring-tailed lemur	Start Frequency (Hz)	End Frequency (Hz)	Peak Frequency (Hz)	Duration (secs)	Source
Female lemur’s (Tellus) 1st call	892	935	928	1.849	Current paper
Female lemur’s (Tellus) 2nd call	797	965	965	0.453	Current paper
DLC Male 1 (Fritz)	1105	944	1046	1.189	Cromer (unpub.)
DLC Male 2 (Licinius)	1192	1154	1171	1.174	Cromer (unpub.)
9 DLC Males’ 110 howls	520–1880 Median 1,040	720–960 Median 880	920–1880 Median 1,160	0.456–1.293 Median 0.762	Macedonia (1993)

Supplemental Fig. 2 shows that the spectrograms of Tellus’s calls have about five (top) and seven (second from top) horizontal striations, respectively, and appear to run across the screen for about 0.5 s (top) and 1.5 s (second from top). The third spectrogram shows that the spectrogram of Male 1’s (Fritz’s) call has about eight horizontal striations, while Licinius has about ten (bottom). Both males’ calls lasted approximately 1.3 s. In addition, the left spectrogram (Suppl Fig. 3) shows about four striations, while the spectrogram on the right has about five striations (Macedonia 1993). Both of these males’ howls last between approximately 0.8 to 1 s (Suppl Fig. 3).

When evaluating whether Tellus’ vocalizations were structurally similar to howls produced by Fritz and other males, the DFA correctly classified 52.27% of the calls to the correct sex. Of the two female howls produced by Tellus, one was classified correctly as a female and the other was classified as a male vocalization. Also, among the male howls, 20 were classified as female. The discriminant function was nonsignificant with a small eigenvalue and low canonical correlation (0.038 and 0.19, respectively). In addition, Wilks’ was not significant (Wilks’ = 096, ^2^ (4, *N* = 39) = 0.377, *p* = 0.8234). Overall, the results of the DFA indicated that Tellus’s calls were acoustically indistinguishable from male howls.

To determine if the overall characteristics of Tellus’s vocalization spectrograms resembled other ring-tailed lemur vocalizations, they were compared to spectrograms of ten ring-tailed lemur vocalizations from Macedonia (1993) - chirp, click, gulp, hmm, howl, huh, late-high wail, moan, twitter, and yip. The shape of Tellus’s two howl spectrograms (Suppl Fig. 2) most resemble Suppl Fig. 4’s howl spectrograms with the striations appearing horizontal. Also, it is clear that visually, the overall shape, structure, and pronounced striations of Tellus’s two call spectrograms (Suppl Fig. 2) most resemble

Macedonia’s (1993) howl spectrogram more than the other nine vocalizations.

## Discussion / Conclusion

When considering all of the evidence including previous reports from the field (e.g., Jolly 1966; Sauther 1991), the female Tellus’s body posture, the structural similarity of her calls to the spectrograms of DLC males and previously published data and images, as well as the results of the DFA, we feel confident concluding that Tellus, a female ring-tailed lemur, produced a howl vocalization. Howling is clearly a rare behaviour in females though or it would have been observed by researchers more frequently. Thus, it is entirely possible that female ring-tailed lemur howling could be a capability of only certain - not all - females in specific situations, and not a functional adaptation.

Another explanation could be that all female ring-tailed lemurs can howl but would only do so in extreme circumstances such as giving birth 50 feet away from her group (Sauther 1991) or separated from her partner when other lemurs are alarm calling, as was the case described in this paper. Sexually mature male ring-tailed lemurs howl (Bolt 2013), and they do so at dusk, more during the breeding season, during intergroup encounters, and when they are separated from the group (Bolt 2013; Jolly 1966; Macedonia 1990a, b, 1993; Petter and Charles-Dominique 1979). Male howling appears to communicate a group’s presence and location, is independent of rank (Bolt 2013; Jolly 1966; Macedonia 1993), and functions as mate defense (Bolt 2013). It is possible that howling, for both male and female ring-tailed lemurs, reflects a state of agitation and/or stress. For Tellus, separation from her partner, and the stress caused by the separation, could explain why she howled.

There are several limitations to this study. First, and foremost, the data are from an opportunistic sampling of one individual making two vocalizations in a captive setting. Second, incorporating data from previously published sources potentially leads to compounded variation and errors in measurement that influence the outcome of the statistical analyses. However, the data (albeit limited) indicate that at least some female ring-tailed lemurs can produce howl vocalizations and that their howls resemble the acoustic qualities of male howls. Given how rarely female ring-tailed howling has been observed in the wild and that it has never previously been recorded, these data provide an important contribution. The last comprehensive survey of the ring-tailed lemur vocal repertoire was more than 30 years ago by Macedonia (1993). Given the results of this study, ring-tailed lemur vocalizations need to be revisited to better account for sex-specific vocal patterns and contexts, both in captive and natural settings. Advanced acoustic recording and analytical tools may help elucidate a more comprehensive understanding of ring-tailed lemur vocal communication.

## Supplementary Information

Below is the link to the electronic supplementary material.

Supplementary Material 1
