# Supplementary material for: First record of an adult female ring-tailed lemur (Lemur catta) making a male-specific howl vocalization
Source: Primates. 2026 Jul 14;67(6):603–8. doi: 10.1007/s10329-026-01270-3 (PMC13731687; doi:10.1007/s10329-026-01270-3)
Supplement: Supplementary file 1 [file 10329_2026_1270_MOESM1_ESM.docx]

**Female Ring-tailed Lemurs Howl Supplementary Material**

Figure 1. Photos of female Tellus (Figure 1A) and Male 1 (Fritz) (Figure 1B) howling. Note how both lemurs’ bodies are similar and in upright positions with their heads and necks raised upward, their chests puffed out, sitting, and holding a substrate. The images were generated from screenshots of video-recordings.

20

Figure 2. Spectrograms of Tellus, the female’s two calls (on top) and two male howls (on bottom). Tellus’s calls have five (top) and seven (second from top) horizontal striations, respectively, and last .5 seconds (top) and 1.5 seconds (second from top). The third spectrogram is a male’s and has about eight horizontal striations, and the bottom male’s spectrogram has ten. Both males’ calls lasted ~1.3 seconds.

21

Figure 3. Two male howl spectrograms from Macedonia (1993). These spectrograms have four and five striations respectively, and go up to 5 kHz.

Figure 4. Spectrograms of ten ring-tailed lemur vocalizations from Macedonia (1993) - gulp, chirp, twitter, click, late-high wail, hmm, yip, huh, howl, and moan. Note how of all the vocalizations, the overall shape, structure, and pronounced striations of Tellus’s two call spectrograms (Suppl Figure 2) most resemble the howl spectrogram depicted here.
